# P-1576. Examining the Association Between Diagnostic Uncertainty at Time of Admission and Poor Functional Outcomes at Discharge among Patients with Suspected Sepsis across 270 Hospitals

**DOI:** 10.1093/ofid/ofaf695.1756

**Published:** 2026-01-11

**Authors:** Shruthi Ganesh, Chih Chun Tung, Joel Alejandro, Jonathan Baghdadi

**Affiliations:** University of Maryland - Institute for Health Computing, North Bethesda, Maryland; University of Maryland School of Pharmacy, Baltimore, Maryland; University of Maryland Medical Center - Midtown Campus, Baltimore, Maryland; University of Maryland School of Medicine, Baltimore, Maryland

## Abstract

**Background:**

In the context of sepsis, vague presenting symptoms have been associated with worse prognosis. This study sought to evaluate whether diagnostic uncertainty at the time of admission, as represented by admission for symptoms rather than a specific disease diagnosis, was associated with death or decreased functional capacity at discharge.

**Figure:**
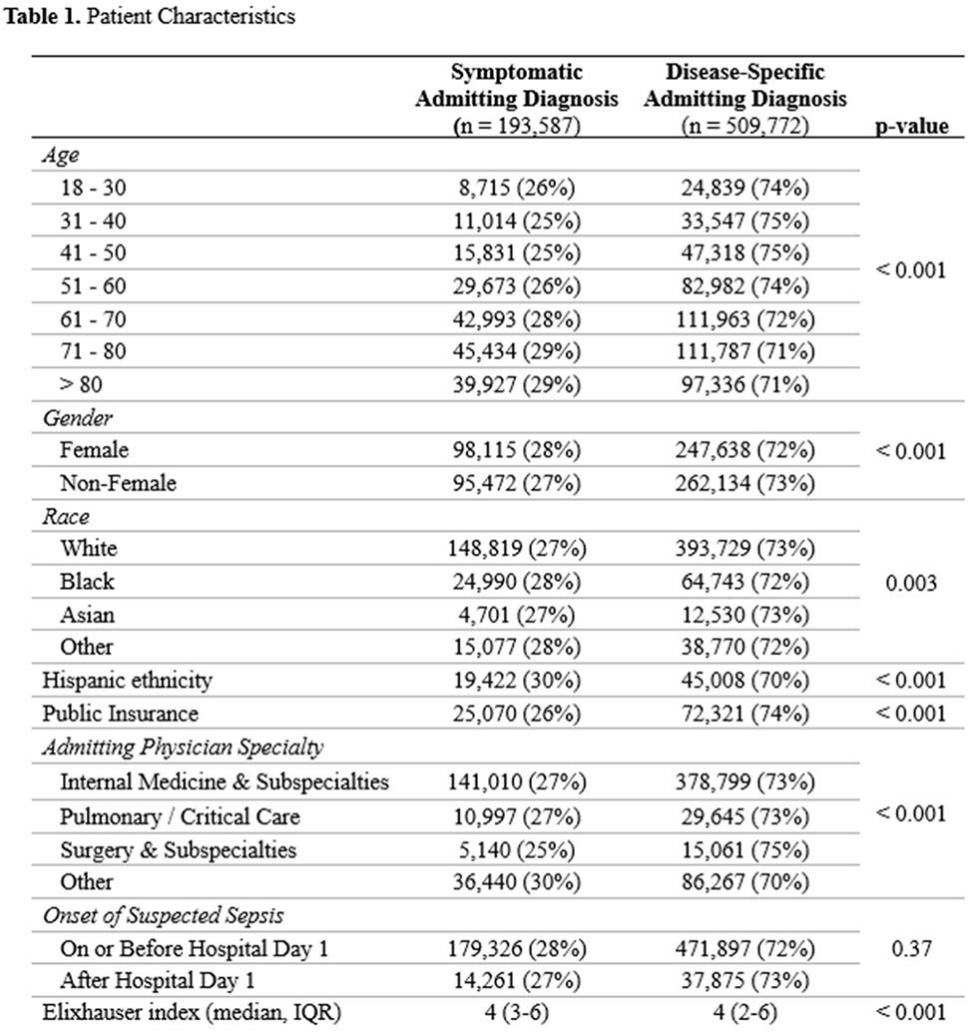

**Figure:**
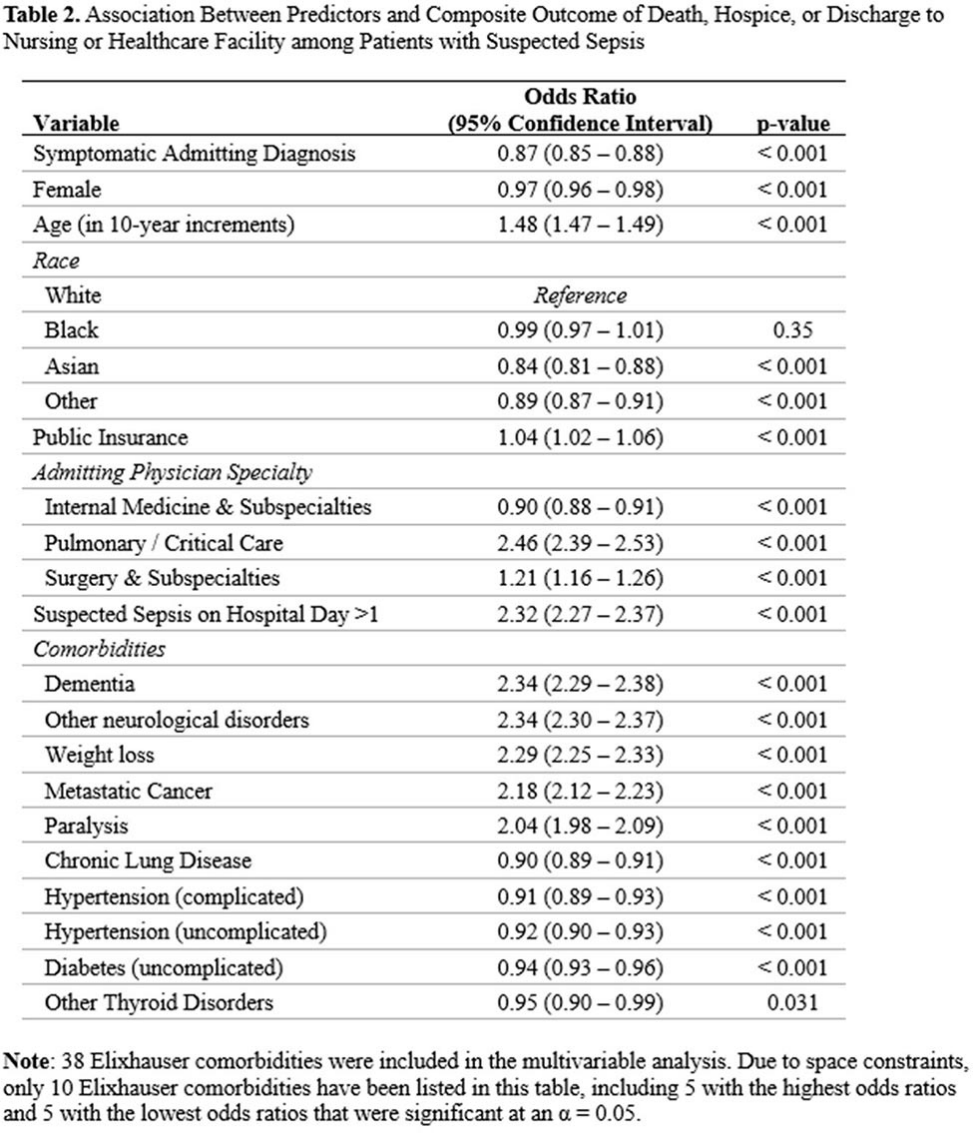

**Methods:**

We conducted a retrospective cohort study of adult inpatients with suspected sepsis from 2019-2023 using the PINC-AI database. Suspected sepsis was defined by a single hospital day in which blood cultures were collected, serum lactate was measured, and IV antibiotics were administered. Symptomatic admitting diagnoses, such as fever or altered mental status, were identified using the “SYM” body system in the AHRQ Clinical Classification Software Refined. The primary outcome was a composite of death, hospice, or discharge to a nursing or healthcare facility. Only patients admitted emergently from home or clinic were included. Multilevel multivariable logistic regression with random effects for hospital was used to evaluate the association between predictors and the composite outcome.

**Results:**

703,359 patients with suspected sepsis across 270 hospitals were included. The average age was 65 (sd 17), 49% were women, and 28% had a symptomatic admitting diagnosis. Thirty-three percent experienced the composite outcome, including 6% of the cohort who died, 6% discharged to hospice, and 21% discharged to a nursing or healthcare facility. In multivariable analysis, symptomatic admitting diagnosis was associated with decreased likelihood of the composite outcome (OR 0.87, 95% CI 0.85 – 0.88, p < 0.001). Admission to pulmonary / critical care (OR 2.46, 95% CI 2.39 -2.53), development of suspected sepsis after hospital day 1 (OR 2.32, 95% CI 2.27 – 2.37), and baseline dementia (OR 2.34, 95% CI 2.29 – 2.38) were most strongly associated with increased likelihood of the composite outcome.

**Conclusion:**

Among patients with suspected sepsis, admission for symptoms rather than a specific disease diagnosis was associated with reduced likelihood of death or decreased functional capacity at discharge. This finding suggests that the potential negative impact of diagnostic uncertainty at time of admission is outweighed by disease-specific risk factors.

**Disclosures:**

All Authors: No reported disclosures

